# Diode Laser Spectrometer for Diagnostic Assessment of Exhaled Air Components

**DOI:** 10.17691/stm2020.12.5.08

**Published:** 2020-10-28

**Authors:** Ya.Ya. Ponurovsky, A.I. Nadezhdinsky, D.B. Stavrovsky, Yu.P. Shapovalov, M.V. Spiridonov, A.S. Kuzmichev, A.A. Karabinenko, Yu.M. Petrenko

**Affiliations:** Head of the Department of Diode Laser Spectroscopy; Federal Research Center, A.M. Prokhorov General Physics Institute of the Russian Academy of Sciences, 38 Vavilov St., Moscow, 119991, Russia;; Professor, Chief Researcher; Federal Research Center, A.M. Prokhorov General Physics Institute of the Russian Academy of Sciences, 38 Vavilov St., Moscow, 119991, Russia;; Head of the Laboratory of Analytical Measurements; Federal Research Center, A.M. Prokhorov General Physics Institute of the Russian Academy of Sciences, 38 Vavilov St., Moscow, 119991, Russia;; Researcher; Federal Research Center, A.M. Prokhorov General Physics Institute of the Russian Academy of Sciences, 38 Vavilov St., Moscow, 119991, Russia;; Head of the Laboratory of Applied Diode Laser Spectroscopy; Federal Research Center, A.M. Prokhorov General Physics Institute of the Russian Academy of Sciences, 38 Vavilov St., Moscow, 119991, Russia;; Researcher; Federal Research Center, A.M. Prokhorov General Physics Institute of the Russian Academy of Sciences, 38 Vavilov St., Moscow, 119991, Russia;; Professor, Department of Hospital Therapy No.2; Pirogov Russian National Research Medical University, 1 Ostrovitianov St., Moscow, 117997, Russia; Professor, Department of General and Medical Biophysics Pirogov Russian National Research Medical University, 1 Ostrovitianov St., Moscow, 117997, Russia

**Keywords:** diode laser spectroscopy, diode laser gas analyzer, exhaled air components, non-invasive diagnosis

## Abstract

The main requirements for a screening test are simplicity, non-invasiveness, safety of testing procedures, high processing speed, and ability to detect diseases at an early stage. A multichannel gas analyzer for assessment of exhaled air composition (diode laser spectrometer), non-invasive screening, and biomedical testing was developed on the basis of near-infrared diode lasers with fiber output. The device measures the following exhaled air components: ^12^CO_2_, ^13^CO_2_, CH_4_, NH_3_, H_2_O, and H_2_S.

The concentration of molecules was measured in a multi-pass Herriot cell with a reference length of 40 cm, 1.8 L volume, and a total optical path length of 26 m. Three diode lasers manufactured by NTT Electronics (Japan) were used in the work. Detection of CH_4_ was carried out in the 1.65 μm wavelength range, ^12^CO_2_, ^13^CO_2_, and H_2_S levels were measured in the 1.60 μm range, NH_3_ and H_2_O in the 1.51 μm range. All measurements were taken in real time.

Clinical testing of the spectrometer was carried out at V.M. Buyanov City Clinical Hospital of Moscow Department of Health. More than 150 patients were examined. The tests included analysis and measurement of these molecular components in the exhaled air of patients with various diseases. The content of these components was studied in conditions of various changes in the human physiological state (dosed physical activity, relaxation, psychoemotional stress, etc.).

The studies have demonstrated efficacy of using the developed hardware system for assessment of exhaled air components in order to reveal functional disorders in various diseases of the digestive system, cardiorespiratory system, diseases caused by impaired nitrogen-excreting function of the kidneys, etc.

## Introduction

**Figure 1:**
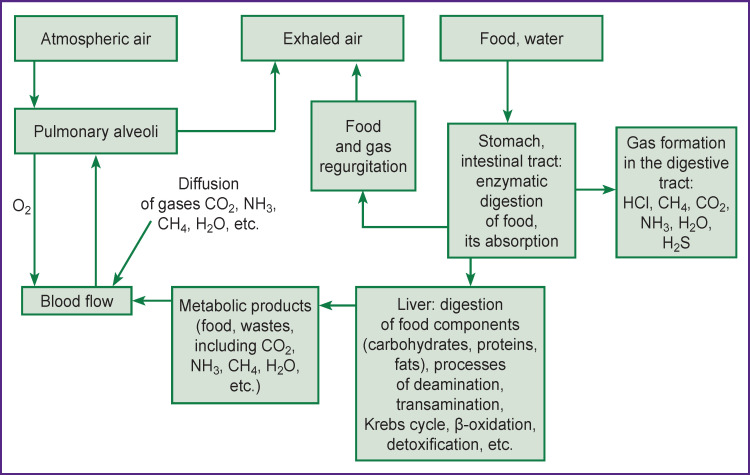
Diagram of exhaled air gas formation

Application of highly sensitive non-invasive methods for analyzing the functional state of the body has recently received much attention in medical diagnostic practice. Analysis of exhaled air (EA) composition is a non-invasive approach based on the characteristics of the volatile components of EA, which in turn reflects the functional state of the lung alveoli, circulatory system, metabolic processes and, consequently, the state of metabolism of the whole body [1, 2]. Our understanding of EA composition is based on the knowledge of physiological and biochemical processes in the human body (Figure 1).

Conventional methods of analyzing gas components in EA include mass spectrometry combined with gas chromatographic separation, electrochemical sensors, ultraviolet chemiluminescence, gas chromatography, infrared (IR) spectroscopy, etc. [3–6]. A characteristic feature of these methods is high selectivity of sampling required to detect microconcentrations of components. However, some of these methods are non-sensitive to changes in nitrogen and oxygen, water and carbon dioxide vapor. Eliminating the influence of background nitrogen and carbon dioxide concentrations is a separate difficult task. Finally, the time required for obtaining the results of EA analysis ranges from tens of minutes to 1.5 h and it is necessary to use expensive permanent equipment in specialized laboratories. Under these circumstances, it is problematic to implement planned screening programs. Application of diode laser spectroscopy for diagnosis of diseases by EA composition is an emerging trend in medicine and biophysics [7, 8].

In Russia, the first studies on measuring the concentration of EA components using diode lasers (DL) were carried out in the early 90s at the General Physics Institute of the Russian Academy of Sciences under the leadership of A.M. Prokhorov, the Nobel Prize winner. Concentrations of carbon monoxide CO and carbon dioxide CO_2_ were measured in the exhalation of smokers using semiconductor lasers in the mid-IR range [9]. Measuring ammonia NH_3_ and methane CH_4_ in the exhalation of healthy and sick individuals was described in [10]. Evaluation of the isotopic ratios of deuterium and hydrogen atoms D/H, oxygen isotopes ^18^O/^16^O and ^17^O/^16^O in water vapor by laser absorption spectroscopy at 2.73 μm wavelength was demonstrated in work [11]. Particular attention was paid to the processing of experimental data on the absorption spectra of water isotopomers and the introduction of adaptive Kalman filtering to improve the measurement accuracy. In study [12], the composition of tobacco smoke was analyzed using a diode laser spectrum analyzer in the mid-IR range. In [13, 14], the concentration of EA ammonia was measured in real time using a quantum cascade laser.

At present, the world DL market is represented by a wide variety of different devices covering a significant spectral range of radiation: from visible light to far-infrared range. Above all, these are lasers for work in the field of fiber-optic communication, spectroscopic studies, gas analysis, for cutting metal, and lasers for ophthalmology, surgery, etc.

Near-IR diode lasers are of particular interest for application in medical diagnostics. They can be several centimeters in size and have radiation power no more than 10 mW, which is safe for eyes. All necessary laser components (active elements, pumps, resonators) are located in one semiconductor crystal.

Nanotechnologies used in the manufacture of DLs provide high efficiency (more than 60%), single-frequency lasing, absence of interference typical for other types of lasers, and allow pre-fabrication of sources with specified properties: the required radiation wavelength, zone of frequency tuning, power, lasing line width, etc. Sensitivity in measuring the absorption of molecules using these DLs is limited only by the quantum noise of laser radiation and makes this laser source a unique tool for spectroscopic studies and gas analysis.

In 2015, a prototype DL gas analyzer (diode laser spectrometer) was developed [15], the first tests were carried out. Subsequently, necessary structural improvements were made and the experimental model of the device, intended for non-invasive screening and biomedical testing was produced. The device is based on near-IR range DL with a fiber output providing the possibility to measure the EA concentrations of isotopic modifications of carbon dioxide ^12^CO_2_, ^13^CO_2_, as well as CH_4_, NH_3_, water vapor H_2_O, and hydrogen sulfide H_2_S, which determines its use as an analyzer of human metabolic functions.

## Technical aspects of the hardware system

**Figure 2:**
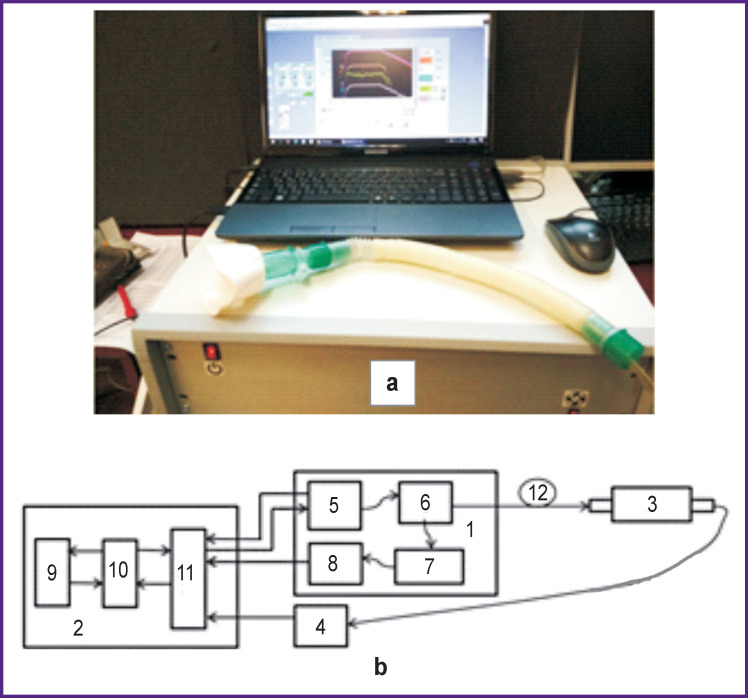
External view (a) and block diagram of a laser channel of the diode laser gas analyzer (b): (*1*) laser radiation unit; (*2*) control, reception, and data processing unit; (*3*) analytical cell with fiber-optic input; (*4*) analytic signal detector; (*5*) diode laser module; (*6*) fiber splitter; (*7*) comparison cell and Fabry–Pérot interferometer; (*8*) comparison signal detector; (*9*) digital programmable module; (*10*) conversion module (DAC and ADC); (*11*) analog signal converter; (*12*) optical fiber cable

The diode laser gas analyzer consists of three laser channels with 1.65 μm wavelength for CH_4_; 1.60 μm — for ^12^CO_2_, ^13^CO_2_, H_2_S; 1.51 μm — for NH_3_, H_2_O. The block diagram of a laser channel is shown in Figure 2 (b).

We used diode laser modules manufactured by NTT Electronics (Japan) [16]. Detectors of analytical signal (*4*) and comparison signal (*8*) are InGaAs-based p-i-n-photodiodes with an active area of 2 mm in diameter [17]. Digital programmable module (*9*), DAC and ADC (*10*) are implemented on the basis of the NI USB-6363 control board (National Instruments, USA). Sampling rate is 2.68 MHz, capacity — 16 bit [18]. Reference length of analytical multi-pass Herriot cell is 40 cm, volume is 1.8 L. The total optical path length is 26 m. The total optical loss is less than 5 dB. The cell is equipped with the DMP 331i pressure sensor (BD SENSORS, Germany) [19]. A mini-compressor with 10 l/min capacity is used to pump EA from the sample to the chamber. Technical characteristics of the DL-gas analyzer are shown in the Table.

**Table:** Diode laser gas analyzer specifications

Parameter	Parameter value
Registration wavelength (nm)/detection limit (ppm):	
СН_4_	1652/0.1
NH_3_, H_2_O	1512/0.03, 100.0
^12^CO_2_, ^13^CO_2_, H_2_S	1602/20.0, 20.0, 0.4
Diode laser frequency stability (cm^–1^)	Less than 0.0002
Diode laser power (mW)	No more than 10
Power consumption (W)	140
Time for setting the operating mode (min)	10
Dimensions W×H×L (mm)	400×300×500
Weight (kg)	22.0
Supply voltage (V)	230
Frequency (Hz)	60

## Algorithm of measuring the concentration of exhaled air components

To reduce the influence of various technical vibrations and electrical noise, to ensure high sensitivity when measuring the concentration of EA components, we used the algorithm based on the amplitude modulation of DL pump current [20]. The procedure of calculating the concentration using this algorithm significantly limits various low-frequency noises in the analytical channel, including the non-selective spectral background due to absorption of gases containing heavy organic EA matter in the tuning range of the laser. Absolute calibration of concentrations is performed at the stage of device installation, using calibration mixtures of the analyzed gases; further calibration is not required. The measurement time for the EA sample is less than 30 s [21].

To illustrate the possibilities of using the developed device, here is the data of studies carried out at V.M.  Buyanov City Clinical Hospital of Moscow Department of Health and the Central Clinical Hospital of the Russian Academy of Sciences. Analysis and measurement of ^12^CO_2_, ^13^CO_2_, CH_4_, NH_3_, H_2_O, and H_2_S components in EA were performed in 152 subjects: 22 healthy volunteers aged 19–31 years and 130 patients aged 19–78 years (75 males, 55 females) with various internal diseases, in a satisfactory condition, with no signs of decompensation of chronic pathologies [22].

The study was carried out in accordance with the Declaration of Helsinki (2013) and approved by the Ethical Committees of V.M. Buyanov City Clinical Hospital of Moscow Department of Health and the Central Clinical Hospital of the Russian Academy of Sciences. Informed consent was obtained from each patient.

**Figure 3:**
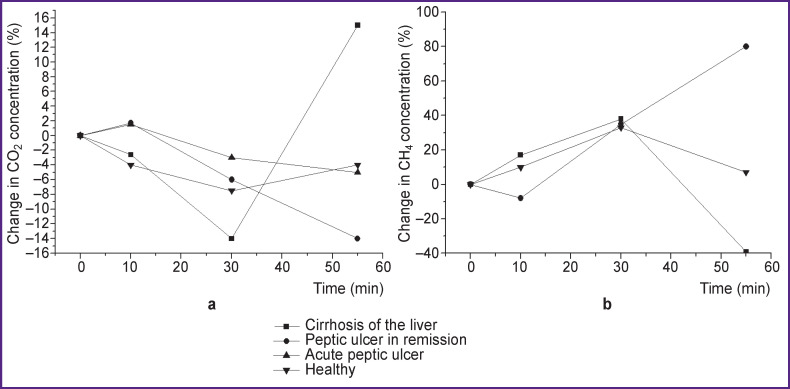
Urea breath test: (a) change in CO_2_ concentration in the exhaled air; (b) change in CH_4_ concentration in the exhaled air

Dynamic assessment of the functional state of the subjects was controlled using standard methods accepted in clinical and physiological studies: measurement of blood pressure, heart rate, respiratory rate, etc. Four groups have been formed (Figure 3). We used the urea breath test for the study, taking into account decomposition of carbamide into NH_3_ and CO_2_ under the influence of urease.

Figure 3 (a) shows the dynamics of the CO_2_ content in EA measured an hour after taking a standard dose of carbamide (500 mg). The most significant changes in CO_2_ concentration were found to manifest in patients with subcompensated liver cirrhosis as compared to other patients. It is likely to be associated with metabolic disorders in these patients.

Figure 3 (b) shows changes in CH_4_ concentration in EA of patients with various pathologies during the urea breath test in dynamics — 10, 30, and 55 min after oral administration. It can be seen that the CH_4_ content in EA is different depending on the nature of the pathological process.

**Figure 4:**
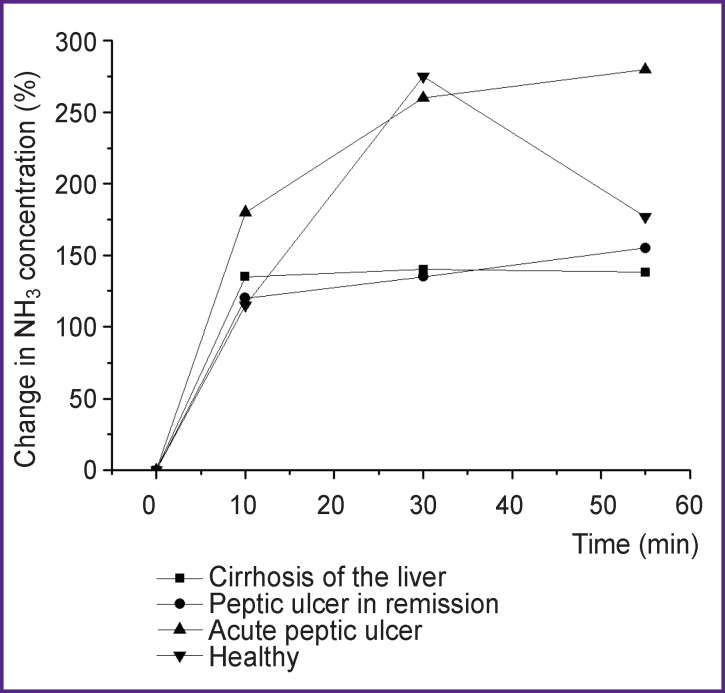
Change in NH_3_ concentration

The dynamics of NH_3_ concentration in EA for different pathologies is shown in Figure 4.

The given graphical characteristics of the investigated gaseous EA components changing under the influence of ammonia or carbamide show the possibility of revealing their dynamics depending on the presence of a gastrointestinal pathology, which may have an important differential diagnostic value. The method of diode laser spectroscopy using the described experimental device offering the possibility to assess the dynamics of components is effective, non-invasive, and safe for wide use in clinical and functional assessment. There have been no side effects in patients during the study.

## Conclusion

The experimental prototype of a diode laser spectrometer for non-invasive screening and biomedical testing based on a near-infrared diode laser allows measuring the concentration of the following exhaled air components: ^12^CO_2_, ^13^CO_2_, CH_4_, NH_3_, H_2_O, and H_2_S. The device offers the possibility to perform real-time analysis of a wide range of exhaled metabolites at rest, especially, when used in combination with various types of exposure indicating metabolic disorders. Analysis carried out using the device is non-invasive and reproducible. Performed online, it significantly reduces examination time. All of this allows us to consider the presented gas analyzer promising for screening as well as clinical and functional assessment.
